# Self-Care Behaviour of Patients With Breast Cancer in the Management of Side Effects of Chemotherapy

**DOI:** 10.7759/cureus.44586

**Published:** 2023-09-02

**Authors:** Beena Koshy, Seetha Lakshmi Avudaiappan, Aravindh S Anand

**Affiliations:** 1 Medical Surgical Nursing, Government College of Nursing, Thiruvananthapuram, Thiruvananthapuram, IND; 2 Medical Surgical Nursing, Sri Ramachandra Institute of Higher Education and Research, Chennai, IND; 3 Miscellaneous, Sri Ramachandra Institute of Higher Education and Research, Chennai, IND; 4 Radiation Oncology, Government Medical College, Thiruvananthapuram, Thiruvananthapuram, IND

**Keywords:** side effects, chemotherapy, breast cancer, self-care behaviour, self-care

## Abstract

Aim

The breast is the leading site of cancer among females. Chemotherapy is the standard treatment of breast cancer and it results in multiple side effects. Apart from pharmacological management, self-care behaviors may significantly influence the management of these side effects. The study aimed to assess the self-care behavior of patients with breast cancer in the management of side effects of chemotherapy.

Methods

A longitudinal descriptive study was conducted at the Daycare chemotherapy unit of the Radio Therapy Department, Government Medical College Hospital Thiruvananthapuram, Kerala, India. In total, 170 female patients with breast cancer receiving their first cycle of chemotherapy participated in the study. Self-care behavior was the primary outcome variable of the study. Sociodemographic and clinical data were measured by using a structured questionnaire. The severity of side effects and self-care behaviors for their management were measured by a Self Care Diary (SCD).

Result

A total of 170 with breast cancer receiving chemotherapy participated in the study. The severity of side effects and self-care behaviors for their management were measured at two separate time intervals, T1 and T2. Severe fatigue was found in 80.0% of participants at T1 and 61.2% at T2. Vomiting (47.7%), mucositis (48.2%), and nausea (49.1%) were also found to be severe at T1, but these side effects were moderate (53.8%, 58.8%, and 51.8% respectively) at T2. Despite the high number of self-care behaviors used to prevent infection, nausea/vomiting, bleeding, decreased appetite, sleeping difficulty, and constipation, overall self-care behaviors were found to be poor. A negative correlation was found between self-care behavior and side effects of chemotherapy.

Conclusion

The study revealed a negative correlation between self-care behaviors and side effects. This indicates that supportive education and training should be given to patients and caregivers to enhance the self-care behaviors of patients to manage the side effects.

## Introduction

Cancer is a group of diseases characterized by uncontrolled and unregulated growth of cells. According to the Globocan 2020 Report, breast cancer is the most common cancer globally. Around 2.3 million (11.7%) cases of breast cancer were registered in 2020 and 0.685 million (6.9%) deaths were reported [1]. As per the Global Cancer Burden and Strategies for Cancer Control (GLOBOCAN) 2020 data, in India, breast cancer accounted for 13.5% (178361) of all cancer cases and 10.6% (90408) of all deaths with a cumulative risk of 2.81 [2]. As per the Hospital Based Cancer Registry (HBCR) report of Regional Cancer Centre, Thiruvananthapuram for the years 2020-2021, it has been found that breast cancer has become the most prevalent cancer in Kerala, accounting for a staggering 32.6% of cases reported [3].

The mortality rate of breast cancer is decreased due to early screening and advanced medical therapies. Treatment of breast cancer is based on the type and its stage. Other factors include the overall health of the patient, menopause status, and personal preferences. The diagnosis of breast cancer and its treatment with surgery, systemic therapy (anticancer chemotherapy, hormonal therapy, targeted therapy, and immunotherapy), and radiation therapy, are associated with significant adverse influences on physical health, mental health, quality of life, and economic status of the patient and family [4].

Chemotherapy is a standard modality of cancer treatment that uses chemical agents or drugs to destroy cancer cells in the cell cycles and inhibit cancer cells’ growth and spread. Chemotherapy drugs can cause side effects, depending on the type and dose of drugs given, and the length of treatment. Chemotherapy has many side effects and serious complications that affect the physical, psychological, social, and spiritual dimensions of an individual’s life and alter their functioning for many months or even years. Therefore, patients need information related to chemotherapy treatment and schedule, the potential side effects, and self-care needs. The cooperation of multidisciplinary health team members and the involvement of both patients and family members in home care is essential to maintain the continuity of care [5].

Neutropenia was the most common chemotherapy-induced hematological toxicity (29.5%), and nausea/vomiting (10.1%) was the most frequent GI toxicity among breast cancer patients [6]. In another study, the most common side effects of chemotherapy for breast cancer were fatigue (82%), followed by constipation (76%), diarrhea, pain, rashes (74%), mucositis (72%), and vomiting (67%) [7]. An increase in the level of knowledge on chemotherapy’s adverse effects would improve the self-care practices in managing the side effects of chemotherapy among cancer survivors, r = 0.55 (p = 0.0010) [8]. Self-management of the side effects of chemotherapy by patients with breast cancer may influence their self-care behavior and quality of life [9].

Patients need to have the necessary knowledge and abilities to set priorities and manage their illness [10]. According to the assumptions of Dorothea Orem’s theory, individuals should be independent and responsible for their care and their family members should assist them if they need it [11].

A cross-sectional descriptive correlational study done in China reported the mean (SD) of uncertainty 76.70 (13.55) on a scale of 28 to 140, self-efficacy 27.15 (5.67) on a scale of 10 to 40, and self-care behaviors 53.96 (6.08) on a scale of 15 to 75 in women receiving chemotherapy for breast cancer [12].

Self-management is essential for improving the quality of life for people with breast cancer. The trajectory of self-care behaviors differs among patients with different psychological and demographic characteristics as therapy progresses [13]. Another study revealed that the self-care practices during each cycle of chemotherapy had improved self-efficacy and performance status to a high level, reducing the side effects of chemotherapy [14].

The finding of RCT done in Urmia concluded that peer education is effective in improving the self-care behavior of patients undergoing cancer chemotherapy (p<0.05) [15]. The intervention group had significantly greater improvements in quality of life status (P < 0.05). Self-care education resulted in a significant increase in the quality-of-life score related to physical (P = 0.00), psychological (P = 0.00), social (P = 0.00), and emotional (P = 0.00) dimensions [16]. An observational cross-sectional study demonstrated that participants had experienced multiple side effects during chemotherapy for breast cancer, but the practice of self-care strategies was only 48%. Age, stage of the disease, and number of experienced side effects significantly affected self-care behavior [17].

Rakhshani et al. proved that there was a significant difference in the mean self-care ability scores of patients undergoing chemotherapy between experimental and control groups after the educational intervention based on Orem’s self-care model [18]. Another study reported the lowest healthy lifestyle behavior scores for physical activity and the highest scores for spiritual growth in women diagnosed with breast cancer receiving chemotherapy. Patients with anxiety had lower spiritual growth and interpersonal relation scores [19]. Face-to-face psychoeducational intervention for patients with breast cancer has been shown to be effective in improving knowledge, resilience, and quality of life during and after chemotherapy [20]. A significant relationship was established between self-management behavior, relaxation, and psychological, physical, and spiritual perceived well-being by having (p = 0.01) in all three parameters [21].

A literature review showed that health literacy is associated with critical self-management behaviors in patients with cancer [22]. A longitudinal quasi-experimental study reported that self-care education significantly improved the activation level (p≤0.001), psychological distress (anxiety level (p≤0.001), depression level (p≤0.001)), and treatment-related concerns regarding symptom management during chemotherapy for breast cancer [23].

Although there are several studies available on the self-care of patients undergoing chemotherapy in various countries, there is not much data regarding the same in India, especially in Kerala. The cancer cases and the number of patients undergoing chemotherapy are increasing tremendously in this state. So, the purpose of the present study is to add some body of knowledge regarding the self-care behavior of patients having breast cancer to deal with chemotherapy-related adverse events.

## Materials and methods

Study design and setting

A longitudinal descriptive study was conducted at the Daycare chemotherapy unit of the Radio Therapy Department, Govt. Medical College Hospital Thiruvananthapuram, Kerala, India from June 2020 to November 2021.

Study sample and sampling

In total, 170 female patients with clinically diagnosed breast cancer (stage I, II, or III) above the age of 18 years with an ECOG (Eastern Cooperative Oncology Group) performance status less than or equal to 2 (ambulatory and capable of all self-care and with more than 50% of waking hours), receiving combination chemotherapy (more than one chemotherapeutic agents with cyclophosphamide, 5-fluorouracil, adriamycin or paclitaxel) for the first time were selected consecutively for the study. Patients having previous exposure to chemotherapy, undergoing concurrent radiotherapy, having visual, hearing, or cognitive impairment, and having known psychiatric illness were excluded from the study. 

Research instruments

The following data were collected through structured personal interviews and reviewing the patient’s medical records.

Socio-Demographic and Clinical Characteristics of the Patients

The following socio-demographic and clinical characteristics were collected from the patients: age, gender, marital status, education, occupation, place of residence, type of family, monthly family income, adverse health habits, and family history of cancer. Clinical data included stage of cancer, type of breast cancer, type of chemotherapy (adjuvant/neoadjuvant), chemotherapy drugs prescribed, number of cycles of chemotherapy scheduled, comorbidities, and ECOG performance status [24].

Self-Care Diary (SCD)

The Self-Care Diary (SCD) was developed by Nail et al. to measure the incidence, severity of selected side effects of chemotherapy and the self-care activities used to manage the side effects, and how effective the activities were in managing each side effect [25]. The original SCD consists of 16 side effects and the self-care activities were rated using a five-point scale ranging from “no relief” to “complete relief”. However, the researchers adopted only twelve side effects most commonly reported by breast cancer patients during chemotherapy, based on the literature review. The modified tool is translated to the local language (Malayalam) by a language expert and consistency of English and Malayalam Language was assured by back translation. Content validity was checked by eight experts (two oncologists, one oncology postgraduate nurse, one oncology nursing faculty, and four nursing research experts) and the content validity index was calculated statistically. (Relevance 0.84-0.94, appropriateness 1, and essentiality 1). In this study, the internal consistency of the tool using Cronbach’s alpha was 0.959. 

Ethical consideration

The Institutional Human Ethics Committee approval was obtained before starting the study (CNT/IEC/37/03/19, IEC-N1/19/NOV/71/88). The purpose of the study was explained and written informed consent was obtained from each participant.

Procedure

Formal permission for data collection was obtained from the Research Committee and Human Ethics Committee before the study. Patients attending the Daycare chemotherapy unit to receive the first cycle of chemotherapy for breast cancer were interviewed for the inclusion and exclusion criteria. The investigator approached the participants, explained the nature and purpose of the study, established a rapport, obtained informed consent, and collected the socio-personal and clinical data. The outcome variables of the study (self-care behaviors and the side effects of chemotherapy) were measured at two separate time intervals, T1 (when they came for the second cycle of chemotherapy) and T2 (during the third cycle of Chemotherapy).

Statistical analysis

The collected data were tabulated and analyzed using Statistical Package for Social Sciences (SPSS) software for Windows version 20.0 (IBM Corp., Armonk, NY). Quantitative variables were described by mean and standard deviation. Qualitative variables were described by proportion and percentage. The comparison of outcomes between two separate time periods was analyzed by using the Wilcoxon signed-rank test and the correlation between self-care behaviors and the side effects of chemotherapy were analyzed using the Spearman rank correlation. Observed differences and associations were considered significant (p <0.05); highly significant if p<0.01, and non-significant if p>0.05.

## Results

One hundred seventy women with breast cancer undergoing chemotherapy participated in the study. The results of the study are presented in five tables.

Table 1 shows that the mean age of the participants was 54.7 ± 9.7. Regarding educational status, 51.8% had only a high school education, 53.5% were unemployed, 77.6% were married, 75.9% belonged to low economic category (below the poverty line), 78.8% were residing in rural areas, 83.5% were in a nuclear family unit, 74.7% had no family history of cancer, 49.4% had stage III breast cancer, 84.7% had the invasive type of cancer, 52.4% received adjuvant chemotherapy, 94.7% received chemotherapy cycles with an interval of three weeks, 72.4% had no comorbidities and 85.3% were completely healthy as per ECOG performance status. 

**Table 1:** Baseline characteristics of participants APL: above poverty line, BPL: below poverty line

Sociodemographic and Clinical Variables			
		Count(n)	Percent (%)
Age in years	<=50	57	33.5
	51 - 60	71	41.8
	>60	42	24.7
	Mean ± SD (54.7 ± 9.7)		
Education	Illiterate/primary up to 7	37	21.8
	High school	88	51.8
	Higher secondary	26	15.2
	Graduate/PG/professional	19	11.2
Occupation	Unemployed	91	53.5
	Unskilled worker	50	29.4
	Skilled worker	9	5.3
	Others	20	11.8
Marital status	Married	132	77.6
	Others	38	22.4
Monthly income of the family	BPL	129	75.9
	APL	41	24.1
Area of residence	Rural	134	78.8
	Urban	16	9.4
	Semi-urban	20	11.8
Type of family	Nuclear	142	83.5
	Joint/extended	28	16.5
Family history of cancer	No	127	74.7
	Yes	43	25.3
Stage of breast cancer			
	Stage I	12	7.1
	Stage II	74	43.5
	Stage III	84	49.4
Type of breast cancer	Invasive	144	84.7
	Infiltrating	20	11.8
	Mucinous	6	3.5
Type of chemotherapy	Adjuvant	89	52.4
	Neo-adjuvant	81	47.6
Interval between cycles	2 weeks	9	5.3
	3 weeks	161	94.7
Comorbidities	No	123	72.4
	Yes	47	27.6
ECOG performance status	Completely healthy	145	85.3
	Ambulatory, restricted/only self-care	25	14.7

Table 2 shows the distribution of self-reported side effects at two separate time intervals T1 and T2. 80.0% of participants at T1 and 61.2% at T2 had severe fatigue. Side effects were severe for vomiting (47.7%), mucositis (48.2%), and nausea (49.1%), at T1 and moderate for vomiting (53 .8%), mucositis (58.8%) and nausea (51.8%) at T2. Moderate sleeping difficulty was reported by 56.5% of participants at T1 and 54.2% at T2. A moderate decrease in appetite was reported by 80% of participants at T1 and 73.5% at T2. Taste change was moderate for 87.1% and 84.7% at T1 and T2 respectively. No constipation for 61.8% at T1 and 68.3% at T2. No episode of diarrhea for 99.4% of patients at T1 and T2. Around 100% participants had incomplete hair loss at T1 and 97.1% had complete hair loss at T2.

**Table 2:** Distribution of self-reported side effects

		First interval (T1)		Second interval (T2)	
Side effects	Severity	Count	Percent	Count	Percent
Sleeping difficulty	Nil	1	0.6	0	0.0
	Mild	44	25.9	56	32.9
	Moderate	96	56.5	92	54.2
	Severe	28	16.4	22	12.9
	Very severe	1	0.6	0	0.0
Decreased appetite	Nil	0	0.0	0	0.0
	Mild	4	2.4	25	14.7
	Moderate	136	80.0	125	73.5
	Severe	29	17.0	20	11.8
	Very severe	1	0.6	0	0.0
Constipation	Nil	105	61.8	116	68.3
	Mild	19	11.1	49	28.8
	Moderate	45	26.5	5	2.9
	Severe	1	0.6	0	0.0
Diarrhea	Nil	169	99.4	169	99.4
	Mild	1	0.6	0	0.0
	Moderate	0	0.0	1	0.6
Hair loss	Incomplete	170	100.0	5	2.9
	Complete	0	0.0	165	97.1
Mucositis	Nil	1	0.6	1	0.6
	Mild	8	4.7	6	3.5
	Moderate	76	44.7	100	58.8
	Severe	82	48.2	62	36.5
	Very severe	3	1.8	1	0.6
Numbness	Nil	164	96.5	166	97.6
	Mild	6	3.5	1	0.6
	Moderate	0	0.0	2	1.2
	Severe	0	0.0	1	0.6
Nausea	Nil	0	0.0	0	0.0
	Mild	4	2.4	8	4.7
	Moderate	68	40.2	88	51.8
	Severe	83	49.1	57	33.5
	Very severe	14	8.3	17	10.0
Vomiting	Nil	0	0.0	0	0.0
	Mild	5	2.9	9	5.3
	Moderate	69	40.6	91	53.8
	Severe	81	47.7	52	30.8
	Very severe	15	8.8	17	10.1
Taste Change	Nil	0	0.0	0	0.0
	Mild	14	8.2	23	13.5
	Moderate	148	87.1	144	84.7
	Severe	8	4.7	3	1.8
Fatigue	Mild	0	0.0	1	0.6
	Moderate	10	5.9	33	19.4
	Severe	136	80.0	104	61.2
	Very severe	24	14.1	32	18.8
Fever/infection	Nil	131	77.1	148	87.1
	Mild	3	1.8	1	0.6
	Severe	36	21.1	1	0.6
	Very severe	0	0.0	20	11.7

Table 3 shows the comparison of self-care behaviors in managing the chemotherapy-related side effects between two separate time intervals (T1 and T2). Since the self-care behaviors used for nausea and vomiting were the same, they were combined. There was a significant difference in the mean number of self-care behaviors used for managing sleeping difficulty, constipation, diarrhea, mouth sores (oral mucositis), nausea or vomiting, fatigue, and infection between T1 and T2. Overall the participants used more self-care behaviors in preventing infection (T1, 21.5±4.3; T2, 22.1±4.2), nausea/vomiting (T1, 12.6 ± 3.3; T2, 13 ± 3.6), bleeding (T1, 11 ± 2.3; T2, 11.2 ± 2.1) decreased appetite (T18.9 ± 1.8; T2, 9.2 ± 2.1), sleeping difficulty (T1, 7.7 ± 2.5; T2, 8.6 ± 2.7), and constipation (T1, 8.4 ± 2.1; T2, 8.9 ± 2). 

**Table 3:** Comparison of self-care behaviors for managing the side effects between two-time intervals among patients undergoing chemotherapy for breast cancer Z#, Wilcoxon Signed Rank Test; *, Significant at 0.05 level

Side effects	First interval (T1)		Second interval (T2)		Z#	p
	Mean ± SD	Median	Mean ± SD	Median		
Difficulty sleeping	7.7 ± 2.5	8.0	8.6 ± 2.7	9.0	4.36	p<0.01
Decreased appetite	8.9 ± 1.8	9.0	9.2 ± 2.1	9.0	1.66	0.098
Constipation	8.4 ± 2.1	9.0	8.9 ± 2	9.0	3.29	p<0.01
Diarrhea	5.7 ± 1.7	6.0	5.8 ± 1.3	6.0	2.08*	0.038
Hair loss	3.5 ± 1.4	3.0	3.4 ± 1.4	3.0	0.18	0.858
Sore mouth	6.6 ± 2.1	6.0	6.9 ± 2.2	7.0	2.42*	0.015
Numbness in hands or feet	5.1 ± 2	6.0	5.2 ± 2	6.0	0.89	0.372
Nausea or vomiting	12.6 ± 3.3	13.0	13 ± 3.6	13.0	2.01*	0.044
Taste change	5 ± 1.5	5.0	5.2 ± 1.4	5.0	1.16	0.247
Fatigue	5.3 ± 2.6	5.0	5.7 ± 3	5.0	2*	0.046
Bleeding	11 ± 2.3	11.0	11.2 ± 2.1	11.0	1.91	0.056
Fever or infection	21.5 ± 4.3	21.0	22.1 ± 4.2	21.0	2.01*	0.044

Table 4 shows the self-care behaviors of participants for selected side effects of chemotherapy. From the data, it is clear that the self-care behaviors were very low except for taking stool softeners, intake of properly cooked meat and fish, use of mask and avoidance of bowel straining, and avoidance of contact sports. 

**Table 4:** Self-care behaviors on selected side effects of chemotherapy

Side effects	Self-care behavior	First interval (T1)		Second interval (T2)	
		count	%	count	%
Difficulty sleeping	Got some exercise	1	0.6	3	1.8
	Read or watched TV	19	11.2	18	10.6
	Tried not to think about it	36	21.2	58	34.1
	Avoided TV and Mobile phone before sleep	62	36.5	63	37.1
	Avoided light and sound in the bedroom	35	20.6	45	26.5
	Listened to music just before sleep	6	3.5	19	11.2
Decreased appetite	Ate high-protein foods	10	5.9	13	7.7
	Ate high-calorie foods	10	5.9	15	8.8
	Added supplements to your diet	5	2.9	13	7.7
	Exercised before meals	1	0.6	3	1.8
	Made an extra effort to eat	7	4.1	19	11.2
Constipation	Ate high-fiber foods	8	4.7	8	4.7
	Ate extra fruit	16	9.4	20	11.8
	Got some exercises	1	0.6	3	1.8
	Drank extra fluids	15	8.8	19	11.2
	Took stool softener or laxative	103	60.8	109	64.1
	Walked for half an hour in the morning and evening	2	1.2	3	1.8
Nausea/vomiting	Took prescribed anti-nausea medication	27	15.5	45	26.5
	Rested after meals	10	5.9	9	5.3
	Cleaned mouth teeth more often than usual	8	4.7	6	3.6
	Ate more slowly than usual	6	3.6	8	4.7
	Ate small frequent meals	5	3.0	14	8.2
	Drank clear liquids	11	6.5	16	9.4
	Avoided sweet, fatty, or spicy food	5	2.9	13	7.7
	Exercised more frequently	1	0.6	3	1.8
Bleeding	Ate soft food	6	3.6	6	3.6
	Used soft toothbrush	2	1.2	2	1.2
	Checked mouth every day for gum bleeding	7	4.1	8	4.7
	Checked skin every day for new bruises or petechiae	49	28.9	59	34.7
	Avoided contact sports	139	81.8	144	84.8
	Avoided bowel straining	101	59.4	102	60.6
Fever/infection	Wash hands often	22	13.0	26	15.3
	Avoided contact with people who have infectious diseases such as cold or flu	35	21.6	42	24.7
	Kept fingernails and toenails short and clean	23	13.5	26	15.3
	Frequent mouth wash	2	1.2	3	1.8
	Avoided eating raw fruits and vegetable	27	15.9	26	15.3
	Avoided eating undercooked meat and fish	111	65.3	112	65.9
	Used mask whenever went out	110	64.7	123	72.4
	Took steam inhalation two times daily	2	1.2	4	2.4
	Changed the dress every day after the bath	18	10.6	19	11.1
	Washed genitals with soap and water after toileting or urination	8	4.7	8	4.7

**Table 5:** Correlation between side effects and self-care behaviors at First interval (T1) and Second interval (T2) r, Spearman Rank Correlation; ** Significant at 0.01 level

	r	p	r	p
Difficulty sleeping	-0.525	p<0.01	-0.583	p<0.01
Decreased appetite	-0.466	p<0.01	-0.357	p<0.01
Constipation	-0.571	p<0.01	-0.335	p<0.01
Diarrhea	-0.131	0.089	-0.06	0.438
Hair loss	-0.033	0.665	0.1	0.195
Sore mouth	-0.13	0.092	-0.27	p<0.01
Numbness in hands or feed	-0.077	0.317	-0.065	0.401
Nausea or vomiting	-0.314	p<0.01	-0.431	p<0.01
Taste change	-0.057	0.464	-0.263**	0.001
Fatigue	-0.248**	0.001	-0.513	p<0.01
Bleeding	-0.007	0.925	-0.058	0.452
Fever or infection	-0.473	p<0.01	-0.4	p<0.01

Table 5 shows the correlation between side effects and their corresponding self-care behaviors. A statistically significant negative correlation was found between side effects (difficulty sleeping, decreased appetite, constipation, nausea/vomiting, fatigue, and fever/ infection) and self-care behaviors at the first-time interval (T1) and a negative correlation was found for sore mouth and fatigue with their corresponding self-care behaviors at the second-time interval (T2).

## Discussion

Cancer is a deadly disease of humanity. Chemotherapy treatment is one of the main curative options [26]. The objective of chemotherapy is to stop the growth of abnormally dividing cancer cells. However, normal cells will also be affected by chemotherapy, such as the mucosal lining of the gastrointestinal tract, bone marrow cells, and hair follicles. So, side effects are commonly found in all patients [27]. Adverse effects of chemotherapy may be severe and have a significant impact on the quality of life of patients. Hence patients should be informed about effective self-care strategies to manage adverse effects [28].

The findings of the study revealed that nearly half of the participants were in the age group of 51-60 years with a mean age of 54.7 ± 9.7. This indicates that breast cancer is more common among older age groups. This finding is supported by another study done in Australia, which reported that 65.4% of participants with breast cancer were within the age group 46-65 years [7]. The finding is also congruent with another study done in Taiwan, in which the participants were diagnosed with breast cancer and their mean age was 53.6±9.5years [9]. The severe side effects reported by the participants were nausea, vomiting, mouth sores, hair loss, and fatigue. Other researchers also identified these side effects and they were distressing for the patients [6,29]. The mean number of most frequently used self-care behaviors for the present study were fatigue (5.3), nausea and vomiting (12.6), taste change (5), and mouth sores (6.6). The findings were congruent with an experimental study where the participants in the control group reported a similar mean number of self-care behaviors such as fatigue (4.4), nausea and vomiting (7.7), taste change (3.6), and mouth sores (3.6) [29].

The frequency of the majority of self-care behaviors used by the participants did not change between T1 and T2 (first and second, time intervals). This finding was supported by the finding of another study where the control group used the same self-care behaviors over time [29].

The present study revealed a negative correlation between self-care behaviors and side effects of chemotherapy in patients with breast cancer. The incidence and severity of side effects can be reduced by improving self-care practices. This finding is strongly supported by many studies, where improving self-care practices with education significantly reduced the severity of side effects [15,16,26,29].

The present study revealed that participants used more self-care behaviors in preventing infection, nausea/vomiting, bleeding, decreased appetite, sleeping difficulty, and constipation. Public education and the COVID management protocol, which were prevalent during the data collection period of the current study, may have improved self-care practices to avoid fever and infection. Participants used more self-care behaviors in reducing nausea/vomiting, bleeding, decreased appetite, sleeping difficulty, and constipation. This is supported by the finding of a study where no significant differences were found between the experimental and control groups in a mean number of self-care behaviors used for fatigue, nausea/vomiting, and taste change [29].

Limitations

The technique of data collection was self-reporting, which might have introduced recall bias and affected the accuracy of the data. The scope of the study was limited to the self-care behaviors of patients in managing physical side effects. Tools to measure the psychological well-being of patients were not employed in the study because the instrument used to measure self-care behaviors and side effects (SCD) was too lengthy to collect data from patients receiving chemotherapy in the outpatient department.

## Conclusions

The study identified the self-care behaviors of women to manage the side effects of chemotherapy for breast cancer in South India. Patients experienced multiple side effects during chemotherapy. In the present study, side effects were severe for vomiting, mucositis, and nausea, in the first time interval and moderate for vomiting, mucositis, and nausea in the second time interval. Overall self-care behaviors of patients were poor. The mean number of self-care behaviors used by the participants was high for preventing infection, nausea/vomiting, bleeding, decreased appetite, sleeping difficulty, and constipation. The study revealed a negative correlation between self-care behaviors and side effects. This indicates that supportive education and training should be given to patients and caregivers to enhance the self-care behaviors of patients and to manage the side effects. Experimental studies can be conducted to establish the effectiveness of such patient empowerment strategies.
